# Remote follow-up by pharmacists for blood pressure control in patients with hypertension: a systematic review and a meta-analysis of randomized controlled trials

**DOI:** 10.1038/s41598-024-52894-8

**Published:** 2024-01-30

**Authors:** Noriaki Matsumoto, Tsuyoshi Nakai, Mikio Sakakibara, Yukinori Aimiya, Shinya Sugiura, Jeannie K. Lee, Shigeki Yamada, Tomohiro Mizuno

**Affiliations:** 1 Department of Pharmacotherapeutics and Informatics, Fujita Health University School of Medicine, 1-98 Dengakugakubo, Kutsukake-Cho, Toyoake, Aichi 470-1192 Japan; 2 Sugi Pharmacy Co., Ltd., Obu, Aichi Japan; 3 Department of Pharmacy Practice and Science, The University of Arizona R. Ken Coit College of Pharmacy, Tucson, AZ USA

**Keywords:** Epidemiology, Lifestyle modification

## Abstract

Hypertension is a major cause of cardiovascular diseases. Several recent studies reported that pharmacists’ remote follow-up reduced hypertension patients’ blood pressure (BP). This meta-analysis aims to verify whether remote follow-up by pharmacists improves BP levels and reveal the factors that make the intervention effective. The search, conducted using PubMed/Medline, Embase, and Cochrane Library from June to July 2023, targeted articles published between October 1982 and June 2023, using terms including “pharmacist”, “hypertension”, and “randomized controlled trial (RCT)”. The inclusion criteria were: (a) RCTs involving hypertension patients with or without comorbidities, (b) pharmacists using remote communication tools to conduct follow-up encounter during the intervention period, (c) reporting systolic blood pressure (SBP) at baseline and during intervention. SBP was the primary outcome for the meta-analysis. Thirteen studies (3969 participants) were included in this meta-analysis. The mean difference of SBP between intervention group and control group was − 7.35 mmHg (*P* < 0.0001). Subgroup analyses showed the greater reduction of SBP in the “regularly scheduled follow-up cohort” (− 8.89 mmHg) compared with the “as needed follow-up cohort” (− 3.23 mmHg, *P* < 0.0001). The results revealed that remote follow-up by pharmacists reduced SBP levels in hypertension patients and scheduled remote follow-up may contribute to the effectiveness.

## Introduction

Hypertension is a major cause of strokes and cardiovascular diseases (CVD)^1,2^. Achieving blood pressure goals can dramatically reduce the risks of cardiovascular complications^3–5^. Still, control rates of BP among patients with hypertension remain low (23% for women and 18% for men in 2019)^6^. Suboptimal medication adherence is a well-recognized factor contributing to poor BP control in people with hypertension^7^. Medication event monitoring system^8–10^ and biochemical analysis using chromatography-tandem mass spectrometry^11^ are reported to be useful in detecting nonadherence and improving adherence in research. As biochemical analysis is an invasive method for patients, electronic monitoring is recognized as an easier method; however, neither may be feasible for use in real-world clinical settings. Some meta-analyses support the usefulness of self-monitoring of BP with additional support by professionals. Katrin Uhlig and colleagues revealed that BP self-monitoring plus additional support such as telemonitoring, counseling by healthcare professionals or nonprofessional healthcare coaches, and medication–behavioral management including medication management, physical exercise or dietary management improved BP levels^12^. Self-monitoring of BP worked best when combined with intensive interventions such as systematic medication titration and lifestyle counseling by healthcare professionals^13^. There are some recent reports of pharmacists performing interventions remotely including education and counseling for patients with hypertension. In these reports, follow-up was conducted using a variety of communication tools, including the telephone, web communications, and text messages according to the plan the providers established (e.g., frequency, interval, and timing of contact)^14,15^. A previous meta-analysis revealed that pharmacist interventions with home-based BP telemonitoring improved BP control in chronic kidney disease (CKD) patients^16^. Therefore, self-monitoring and additional support by healthcare practitioners are recognized as methods for improving BP in patients with hypertension. However, it remains unclear which is the most effective support for reducing BP levels. The current study expanded to include patients having hypertension and chronic disease other than CKD and interventions using communication tools in addition to telephones to compare telemonitoring tools and their influence on hypertension outcome during follow-ups. The aim of this meta-analysis was to determine whether remote follow-ups by pharmacists improve BP levels and identify factors that contribute to intervention effectiveness.

## Results

### Study selection

**Figure 1:**
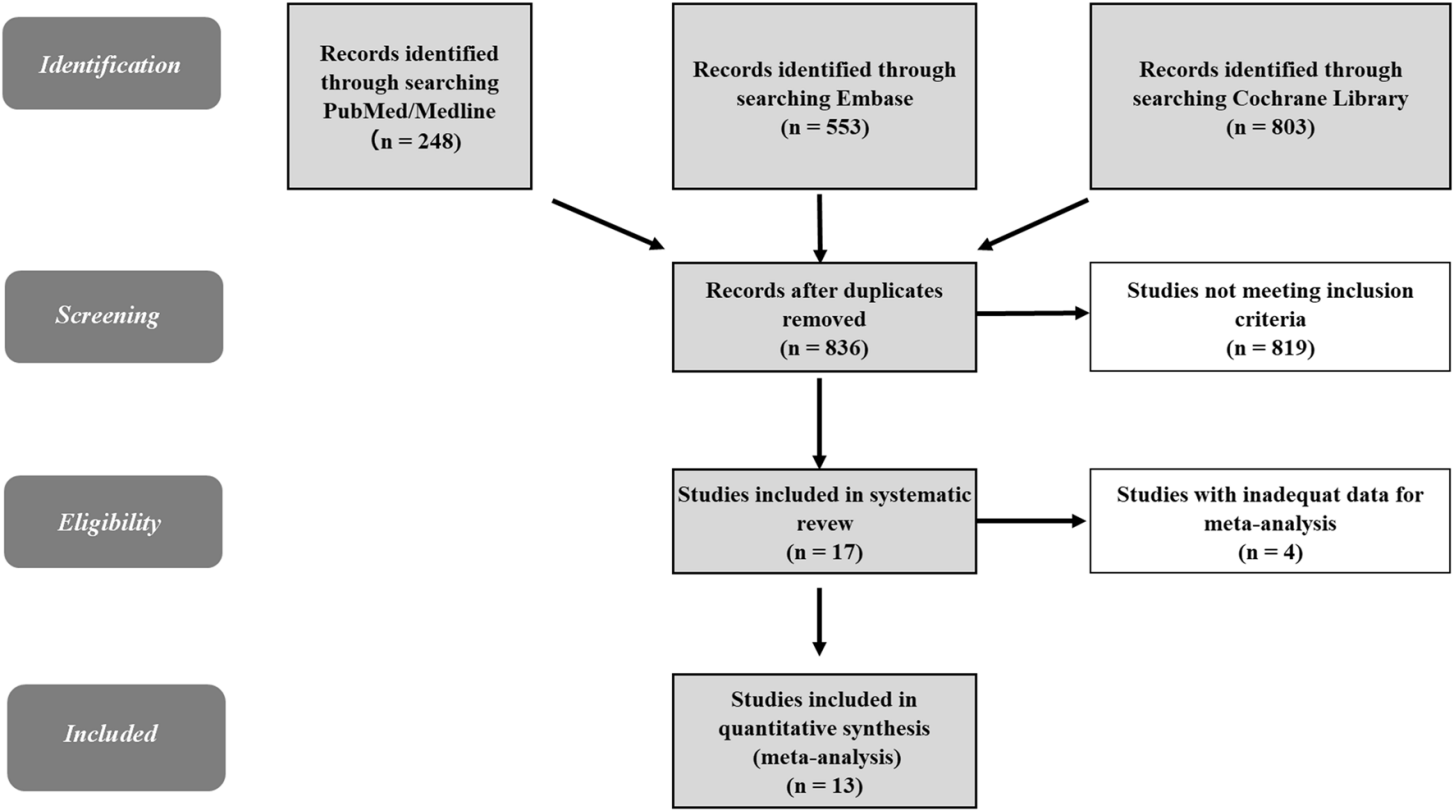
Flow diagram of screening. Duplicate records, non-English records, conference reports, reviews, meta-analyses, research protocols, and reports about non-randomized controlled trial (RCT) trials were excluded from resulting records. The inclusion criteria for the meta-analysis were as follows: (**a**) RCTs involving hypertension patients with or without coexisting chronic conditions, (**b**) pharmacists using remote communication tool(s) to follow up with subjects during the intervention period, (**c**) studies reporting systolic blood pressure at baseline and during the intervention period. In addition, the studies with inadequate values for standard deviation or 95% confidence interval was excluded.

A search of the PubMed/Medline (n = 248), Embase (n = 553), and Cochran Library (n = 803) yielded 1604 relevant studies (Fig. 1) with 836 studies remained after removing duplicates. After excluding non-English records, conference reports, reviews, meta-analyses, research protocols, reports about non-RCT trials, and studies that did not meet the full eligibility criteria, 17 RCTs remained. After further excluding studies with inadequate data, 3969 participants from 13 RCTs were included in the present meta-analysis.

### Study characteristics

Characteristics of 13 studies included in the meta-analysis were summarized in Table 1 in the Supplementary Materials. These 13 studies were conducted in seven countries: USA (n = 6)^14,17–21^, China (n = 2)^22,23^, India (n = 1)^24^, Nigeria (n = 1)^15^, Italy (n = 1)^25^, Australia (n = 1)^26^, Spain (n = 1)^27^. The mean subject age of seven studies was < 65 years^14,15,17,18,20,23,25^, and that of four studies was ≥ 65 years^19,21,26,27^. The mean age was not reported in two articles^22,24^. The intervention period ranged from 8 weeks to 12 months in the 13 included studies.

**Table 1:** Characteristics of studies and patients.

Study author, year	Country	Baseline mean age, years (SD)	Baseline percentage of male patients	Baseline percentage of comorbidities	SBP/baseline (SD or 95%CI)	Intervention period	Tools of communication	Study, n at final point of intervention period	SBP/final point of intervention period (SD or 95%CI)
Solomon DK et al., 1998	USA	IG:66.3(10.0) CG:67.3(11.0)	IG:98.4, CG:92.9	NA	IG:144.4(17.2), CG:146.4(16.3)	6 months	Telephone	IG: 63, CG: 70	IG:138.2 (12.9), CG:144 (20.1)*^3^
Green BB et al., 2008	USA	IG:59.3 (8.6), CG:58.6 (8.5)	IG:44.1, CG:45.3	NA	IG:152.2 (10.4), CG:151.3 (10.6)	12 months	Telephone, web communications	IG: 237, CG: 247	IG:137.9 (136.0,139.8), CG:146.3 (144.5 148.2)
Carter BL et al., 2009	USA	IG:57.3 (14.3), CG:59.2 (13.8)	IG:37.5, CG:44.3	DM, IG: 19.8, CG: 38.1 Heart failure, IG: 0.5, CG: 1.9 Angina, IG: 0.5, CG: 5.7 Peripheral arterial disease, IG: 2.1, CG: 1.9 CKD, IG: 5.7, CG: 7.6 Left-ventricular hypertrophy, IG: 1.6, CG: 1.4	IG:153.6 (12.8), CG:150.6 (14.1)	6 months	Telephone	IG: 192, CG: 210	IG:132.9 (15.5), CG:143.8 (20.5)
Magid DJ et al., 2011	USA	IG:65.1 (11.1), CG: 66.7 (12.2)	IG:66.7, CG:62.8	DM or CKD, IG: 52.2, CG: 58.6	IG:150.5 (19.5), CG:143.8 (16.8)	6 months	Telephone	IG: 138, CG: 145	IG:137.4 (19.4), CG:136.7 (17.0)
Zaragoza-Fernandez et al., 2012	Spain	IG:67.4(9.7), CG:69.3(11.4)	IG:42.1, CG:32.4	DM, IG: 25.0, CG: 28.4 Hyper-Cholesterol, IG: 64.5, CG: 75.7 CVD Antecedents, IG: 32.9, CG: 25.7	IG:147.3(15.1), CG:140.1(9.4)	8 weeks	Telephone	IG: 71, CG: 72	IG:131.6 (13.3), CG:142.0 (10.5)
Margolis KL et al., 2013	USA	IG:62.0 (11.7), CG: 60.2 (12.2)	IG:54.8, CG:55.9	DM, IG: 20.2, CG: 18.0 CKD, IG: 20.6, CG: 16.7 DM or CKD, IG: 35.5, CG: 29.3	IG:148.2 (146.3, 150.0) CG:147.7 (145.8, 149.5)	12 months	Telephone	IG: 197, CG: 191	IG:125.7 (123.4,128.0), CG:134.8 (132.5,137.2)
Stewart K et al., 2014	Australia	IG:66.8 (12.1), CG:66.6 (11.7)	IG:47.8, CG:54.8	CVD, IG: 35.3, CG: 39.4 DM, IG: 19.3, CG: 16.5 Depression, IG: 16.9, CG: 18.1	IG:141.9 (22.4), CG:140.1(22.5)	6 months	SMS, Telephone, Mail	IG: 176, CG: 176	IG:131.7 (22.0), CG:135.3 (22.3)
Carter BL et al., 2015	USA	IG:NA*^1^ CG:61.8	IG:39.2 CG:40.6	DM / kidney disease, IG:NA*^1^, CG: 54.0 No DM / kidney disease, IG:NA*^1^, CG: 46.0	IG:NA*^1^ CG:149.6 (15.3)	9 months*^2^	Telephone	IG: 345, CG: 194*^2^	IG:131.6 (15.8), CG:138.2 (19.7)*^2^
Scala D et al., 2018	Italy	IG:57.5 (10.8), CG:57.7 (12.2)	IG:47.6, CG:50.0	DM, IG: 34.1, CG: 58.8	IG:149.9 (10.3), CG:149.6 (10.0)	12 months	Telephone	IG: 84, CG: 80	IG:135.5 (12.3), CG:147.9 (17.5)
Jackson IL et al., 2021	Nigeria	IG:48.4 (8.8), CG:49.9 (8.8)	IG:39.8, CG:42.7	HIV positive, IG:100, CG: 100 DM, IG: 6.8, CG: 2.9 Peptic ulcer disease, IG: 1.9, CG: 1.9 Renal disease, IG: 10.7, CG: 9.7 Hypertensive heart disease, IG: 1.0, CG: 1.9 Osteoarthritis, IG: 1.9, CG: 3.9 Hemorrhoids, IG: 1.0, CG: 1.9 Benign prostatic hyperplasia, IG: 3.9, CG: 1.0	IG:154.3 (21.7), CG:151.8 (18.1)	12 months	Text messages	IG: 91, CG: 91	IG:137.8 (17.4), CG:148.6 (20.1)
Li Y et al., 2021	China	NA	IG:39.7, CG:45.6	NA	IG:150.61 (20.44), CG:148.34 (17.33)	6 months	Telephone	IG: 290, CG: 298	IG:139.29 (14.53), CG:143.54 (14.12)
Mathews AS et al., 2022	India	NA	IG:38.7, CG:42.3	NA	IG:140.86(5.05), CG:143.00(6.19)	12 months	Telephone	IG: 106, CG: 104	IG:125.81 (4.51), CG:134.78 (5.57)
Li N et al., 2023	China	IG:64.06 (9.43), CG:63.42 (9.06)	IG:66.7, CG:42.0	Coronary heart disease, IG: 56.9, CG: 56.0 DM, IG:45.1, CG: 42.0 Heart failure, IG: 35.3, CG: 40.0 Hyperlipidemia, IG: 54.9, CG: 34.0 Renal insufficiency, IG: 41.2, CG: 34.0 Renal artery stenoses, IG: 19.6, CG: 26.0	IG:144.98 (14.78), CG:143.78 (13.95)	12 months	Telephone	IG: 51, CG: 50	IG:135.51 (9.99), CG:140.14 (10.23)

*SD* standard deviation, *CI* confidence interval, *SBP* systolic blood pressure, *USA* The United States of America, *IG* intervention group, *CG* control group, *NA* not available, *SMS* short message service, *CKD* chronic kidney disease, *CVD* cardiovascular diseases, *DM* diabetes mellitus, *HIV* human immunodeficiency virus.

*^1^IG had been subdivided into two groups with separate baseline.

*^2^Results after 9 months were excluded in the present study because outcomes after 9 months were measured separately for minority and non-minority groups.

*^3^SBP measured at time 2 (about 15 min after arrival of patients to the clinic) was used for the present analysis.

### Effect of remote follow-up for BP control

**Figure 2:**
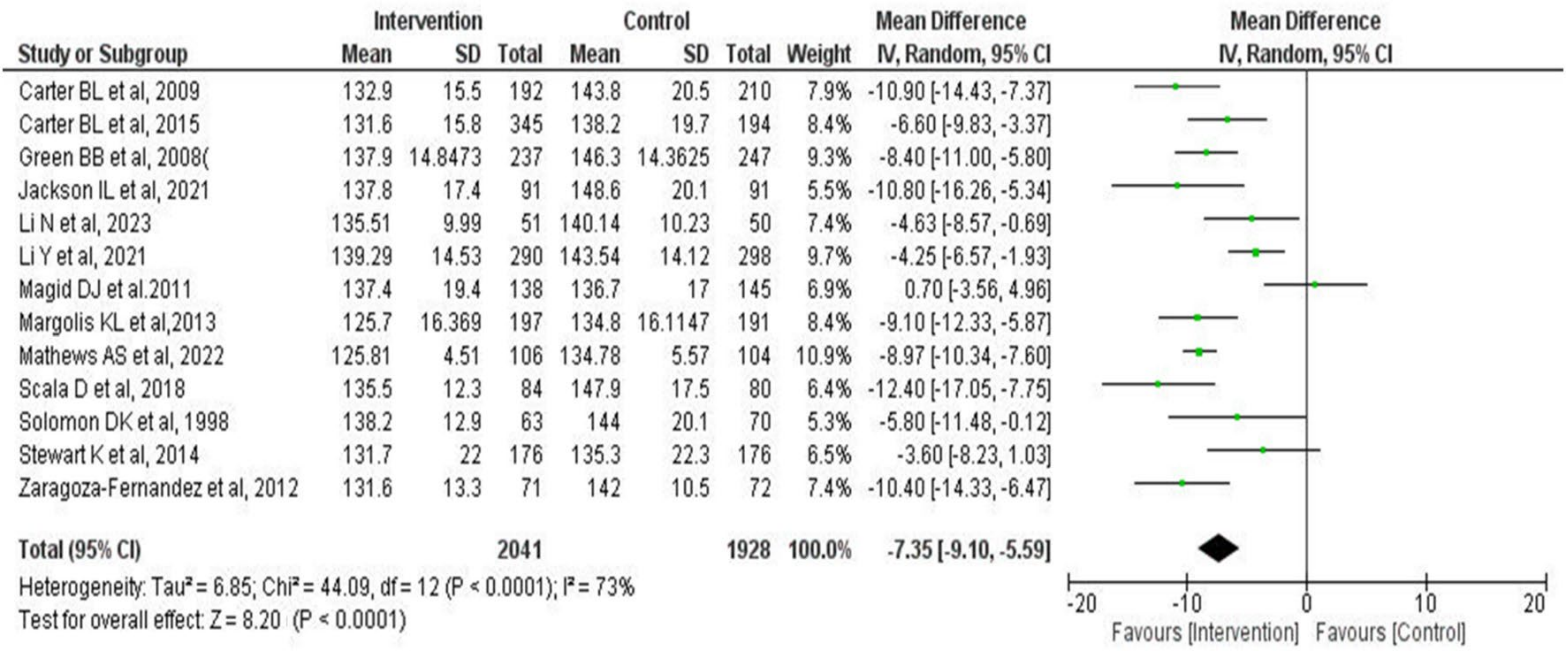
Forest plot of comparison between the intervention and control groups in 13 studies at the final point of the intervention period. A meta-analysis was conducted using the random-effects model. The total number of patients was 3969. The mean difference in SBP between the intervention and control groups was − 7.35 mmHg (95% CI − 9.10 to − 5.59 mmHg, *P* < 0.0001). *SBP* systolic blood pressure, *SD* standard deviation, *CI* confidence interval.

The forest plot of comparison between intervention group (IG) and control group (CG) at the final point of the intervention period in 13 included studies is shown in Fig. 2. The mean difference in SBP between IG and CG was − 7.35 mmHg (95% Confidence Interval [CI] − 9.10 to − 5.59 mmHg, *P* < 0.0001), and there was a high degree of heterogeneity (*χ*^2^ = 44.09, *df* = 12, I^2^ = 73%).

### Subgroup analysis

#### Regularity of follow-up

**Figure 3:**
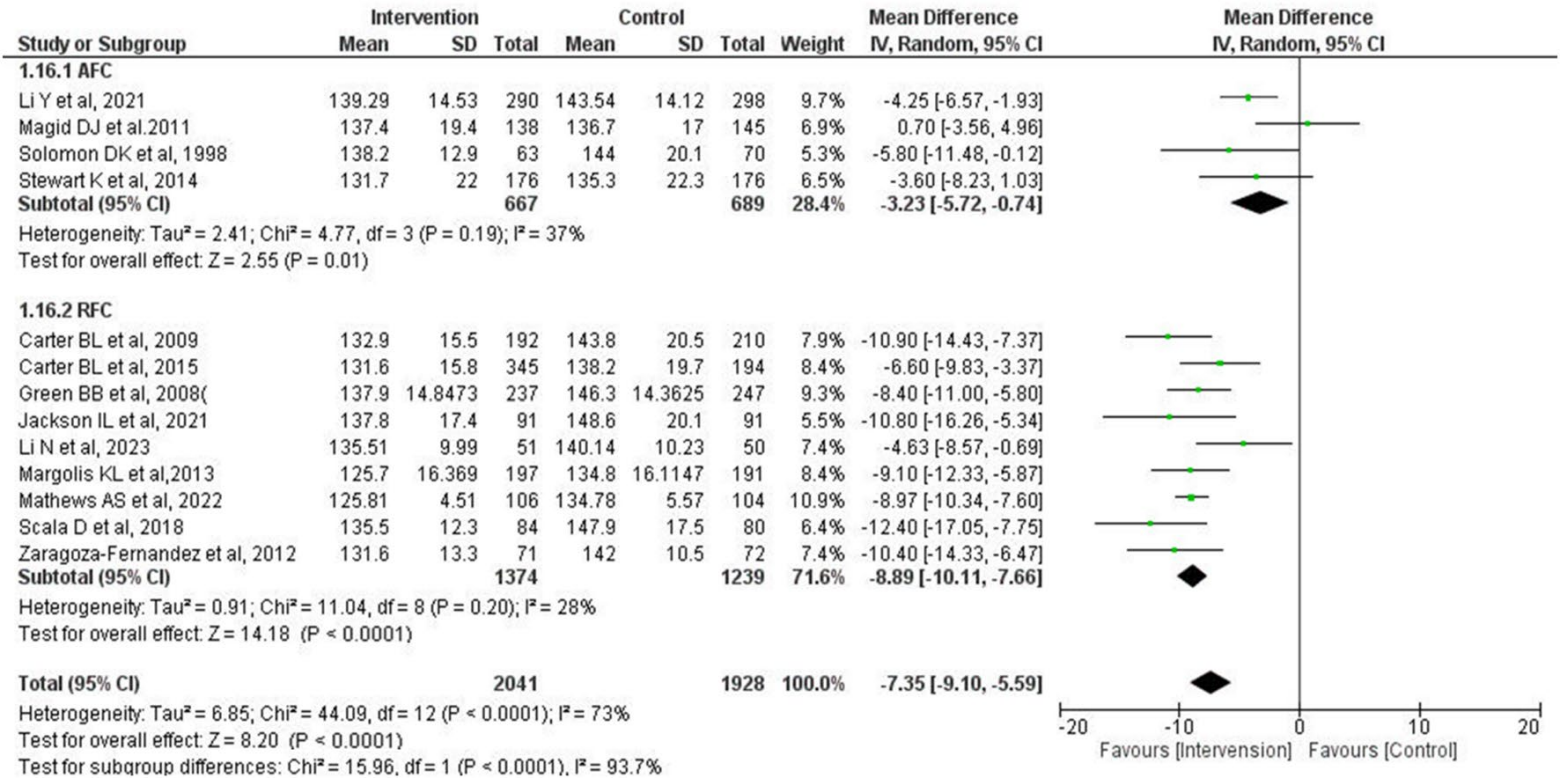
Forest plot of comparison between the “regularly scheduled follow-up cohort” and the “as needed follow-up cohort” at the final point of the intervention period. A meta-analysis was conducted using the random-effects model. The total number of patients and studies was 3969 and 13, respectively. The mean difference of SBP was − 8.89 mmHg (95% CI − 10.11 to − 7.66 mmHg, *P* < 0.0001) and − 3.23 mmHg (95% CI − 5.72 to − 0.74 mmHg, *P* = 0.01) in RFC and AFC, respectively. *SBP* systolic blood pressure, *SD* standard deviation, *CI* confidence interval, *RFC* regularly scheduled follow-up cohort, *AFC* as needed follow-up cohort.

The mean differences in SBP between IG and CG were − 8.89 mmHg (95% CI − 10.11 to − 7.66 mmHg, *P* < 0.0001) and − 3.23 mmHg (95% CI − 5.72 to − 0.74 mmHg, *P* = 0.01) in the “regularly scheduled follow-up cohort (RFC)” and the “as needed follow-up cohort (AFC),” respectively (Fig. 3). In addition, there was a significant subgroup difference between RFC and AFC (*P* < 0.0001). Regarding heterogeneity, significant reductions were observed in both subgroups (*P* = 0.20, I^2^ = 28% in RFC, and *P* = 0.19, I^2^ = 37% in AFC) compared with the overall population (*P* < 0.0001, I^2^ = 73%).

Because there were two studies^19,27^ with large differences in baseline SBP, we repeated the analysis with these two studies removed. Even after excluding these studies, the SBP reduction effect by pharmacist remote follow-up interventions and subgroup differences between RFC and AFC remained significant (Supplementary Fig. 1).

#### Type of communication tools

**Figure 4:**
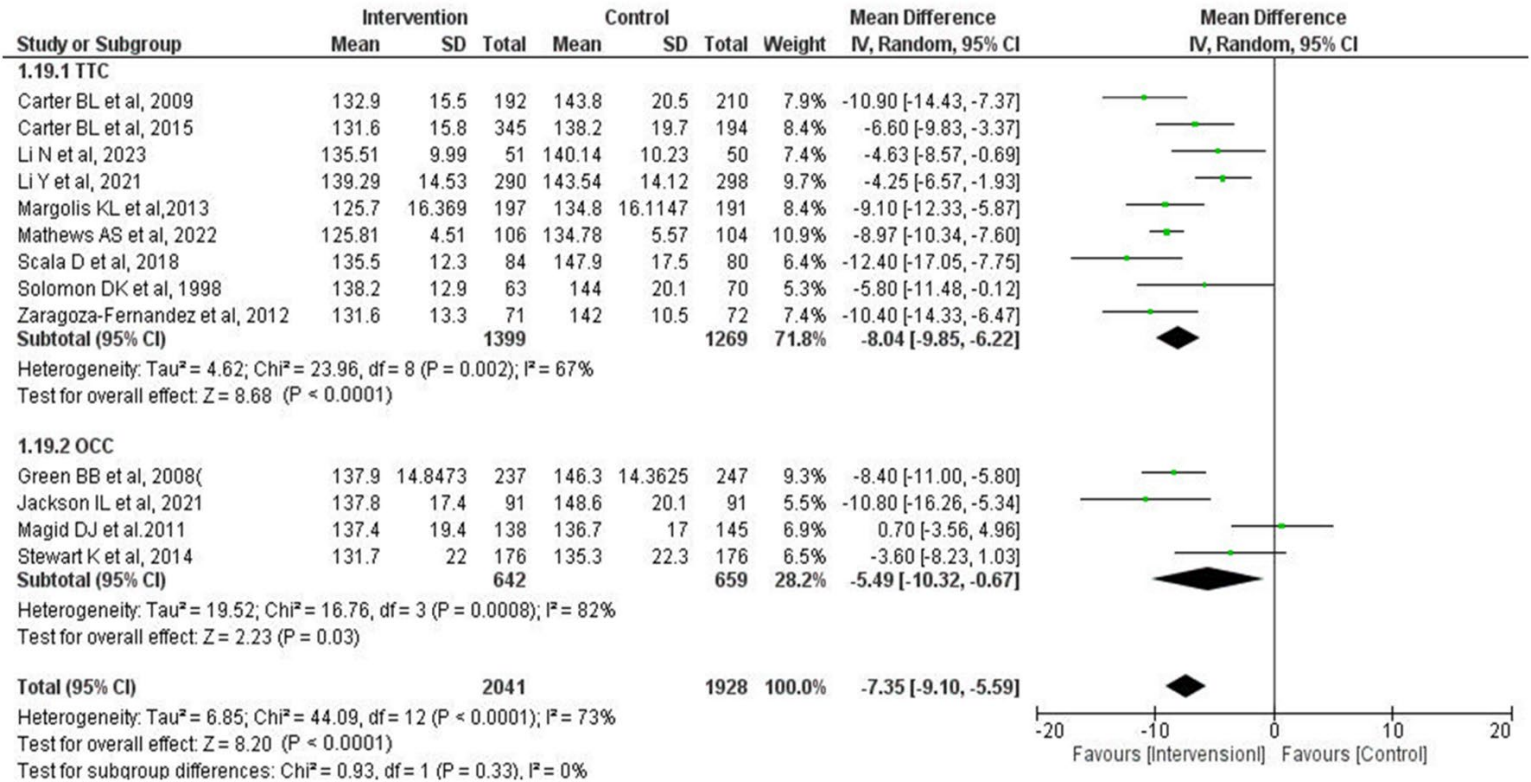
Forest plot of comparison between the “telephone tool cohort” and “other communication tools cohort” at the final point of the intervention period. Meta-analysis was undertaken with random-effects models. The total number of patients and studies was 3969 and 13, respectively. The mean difference of SBP in TTC and OCC was − 8.04 mmHg (95% CI − 9.85 to − 6.22 mmHg, *P* < 0.0001) and − 5.49 mmHg (95% CI − 10.32 to − 0.67 mmHg, *P* = 0.03), respectively. *SBP* systolic blood pressure, *SD* standard deviation, *CI* confidence interval, *TTC* telephone tool cohort, *OCC* other communication tools cohort.

The mean difference in SBP between IG and CG in the “telephone tool cohort (TTC)” and “other communication tool cohort (OCC)” were − 8.04 mmHg (95% CI − 9.85 to − 6.22 mmHg, *P* < 0.0001) and − 5.49 mmHg (95% CI − 10.32 to − 0.67 mmHg, *P* = 0.03), respectively (Fig. 4). There was no significant subgroup difference between TCC and OCC (*P* = 0.33). Regarding heterogeneity, the I^2^ of each subgroup was not significantly different from that of the overall group.

#### Communication with physician

**Figure 5:**
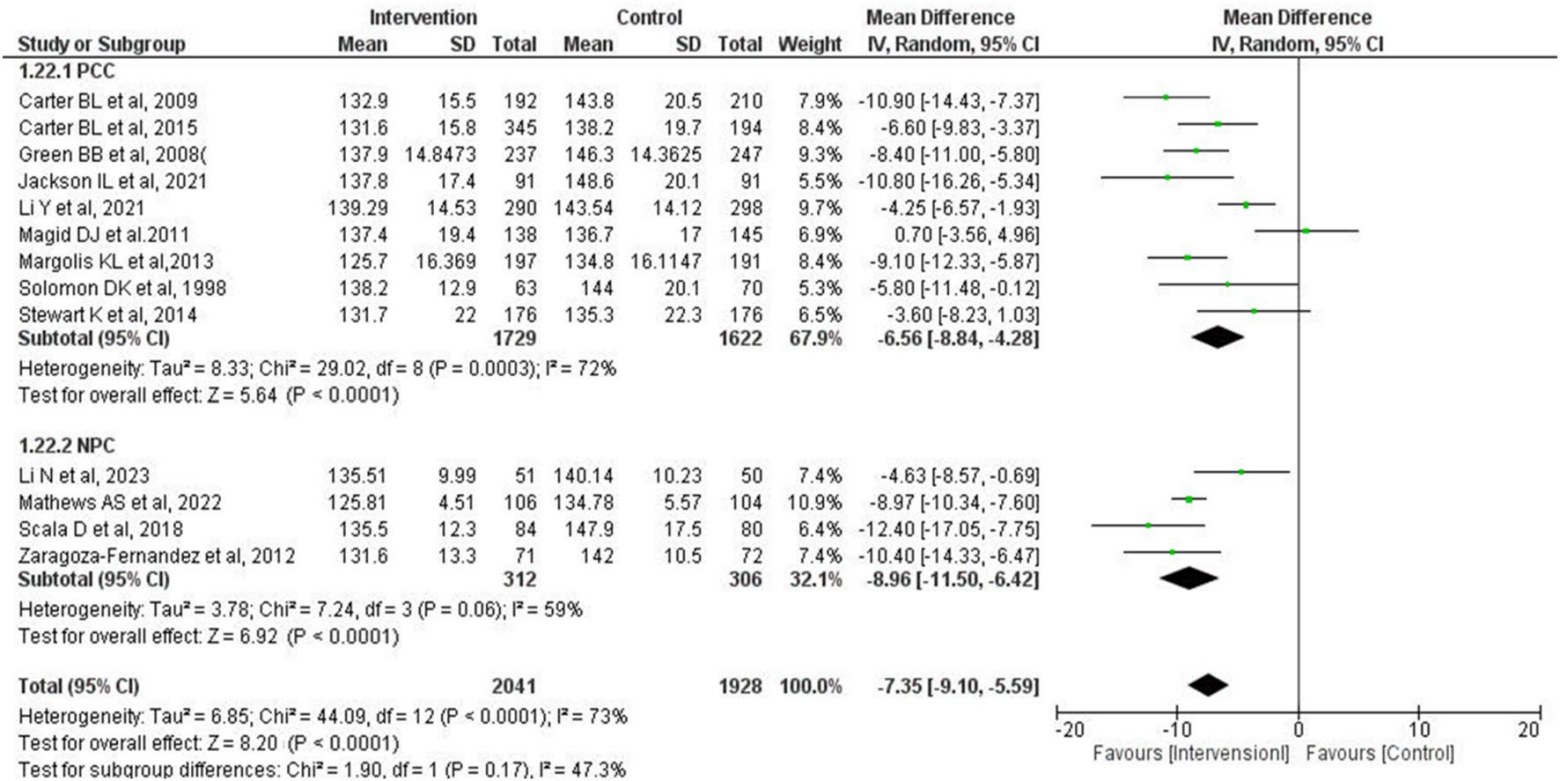
Forest plot of comparison between “physician communication cohort” and “no physician communication cohort” at the final point of the intervention period. (**A**) meta-analysis was conducted using the random-effects model. The total number of patients and studies was 3969 and 13, respectively. The mean difference of SBP in PCC and NPC was − 6.56 mmHg (95% CI − 8.84 to − 4.28 mmHg, *P* < 0.0001) and − 8.96 mmHg (95% CI − 11.50 to − 6.42 mmHg, *P* < 0.0001), respectively. *SBP* systolic blood pressure, *SD* standard deviation, *CI* confidence interval, *PCC* physician communication cohort, *NPC* no physician communication cohort.

The mean difference in SBP between IG and CG in the “physician communication cohort (PCC)” and “no physician communication cohort (NPC)” were − 6.56 mmHg (95% CI − 8.84 to − 4.28 mmHg, *P* < 0.0001) and − 8.96 mmHg (95% CI − 11.50 to − 6.42 mmHg, *P* < 0.0001), respectively (Fig. 5). No significant subgroup difference was observed between PCC and NPC (*P* = 0.17). Regarding heterogeneity, the I^2^ of each subgroup was not significantly different from that of the overall group.

### Risk of bias assessment

The quality of included studies assessed by the Risk of Bias 2 (ROB2) tool is shown in Table 2. Because the number of excluded participants was not described in the study by Solomon and colleagues, it was rated high risk for missing the outcome data^21^. Due to insufficient randomization information and variability in baseline values, the study by Zaragoza-Fernandez et al.^27^ was rated high risk.

**Table 2:** Risk of bias assessment.

Study ID	Randomization process	Deviations from the intended interventions	Missing outcome data	Measurement of the outcome	Selection of the reported result	Overall
Carter et al.^17^	*Some concerns*	Low risk	Low risk	Low risk	Low risk	*Some concerns*
Carter et al.^18^	*Some concerns*	Low risk	Low risk	Low risk	Low risk	*Some concerns*
Green et al.^14^	Low risk	Low risk	Low risk	Low risk	Low risk	Low risk
Jakson and Ukwe^15^	*Some concerns*	Low risk	Low risk	Low risk	Low risk	*Some concerns*
Li et al.^23^	*Some concerns*	Low risk	Low risk	Low risk	Low risk	*Some concerns*
Li et al.^22^	Low risk	Low risk	Low risk	Low risk	Low risk	Low risk
Magid et al.^19^	*Some concerns*	Low risk	Low risk	Low risk	Low risk	*Some concerns*
Margolis et al.^20^	Low risk	Low risk	Low risk	Low risk	Low risk	Low risk
Mathews et al.^24^	*Some concerns*	Low risk	Low risk	Low risk	Low risk	*Some concerns*
Scala et al.^25^	Low risk	Low risk	Low risk	Low risk	Low risk	*Some concerns*
Solomon et al.^21^	*Some concerns*	Low risk	**High risk**	Low risk	Low risk	**High risk**
Stewart et al.^26^	Low risk	Low risk	Low risk	Low risk	Low risk	Low risk
Zaragoza-Fernandez et al.^27^	**High risk**	Low risk	Low risk	Low risk	Low risk	**High risk**

The studies were assessed by following six domains; “Randomization process”, “Deviation from the intended interventions”, “Missing outcome data”, “Measurement of the outcome”, “Selection of the reported result”, and “Overall”.

**Figure 6:**
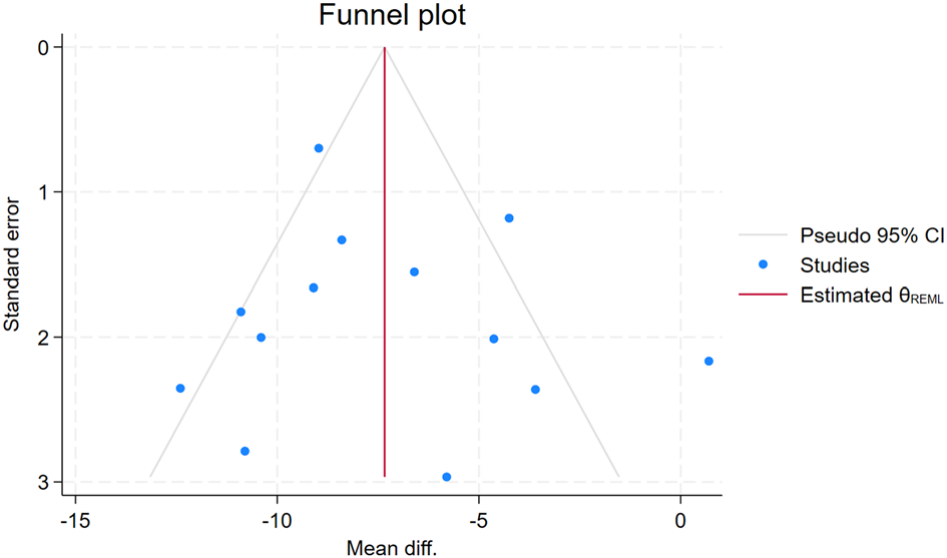
Funnel plot using mean differences of SBP at the final point of the intervention period in 13 studies. *Mean diff.* Mean difference of SBP, *SBP* systolic blood pressure.

A funnel plot was created using mean differences in SBP at the final point of the intervention period in the 13 included studies (Fig. 6). Egger’s test for a regression intercept yielded a *p* value of 0.87, indicating no evidence of publication bias. For the three subgroup analyses, we considered the number of studies in each subgroup to be insufficient to statistically assess publication bias. Result of trim-and-fill analysis is also shown in Supplementary Table 1. There was no sign of publication bias (Mean differences of SBP and 95% CI were slightly different between using Review Manager and using Stata).

### Sensitivity analysis

Results of sensitivity analyses are shown in Supplementary Table 2. The results were not substantively different under any conditions examined.

## Discussion

Because of the recent COVID-19 pandemic, the attention to remote patient encounters and follow-ups has increased more than ever. The present study revealed that remote follow-up by pharmacists improved SBP levels compared with usual care in patients with hypertension. In the intervention group receiving pharmacists’ remote follow-up, regularly scheduled follow-up was found to be an important factor in the success of the intervention compared with as needed follow-up. A previous meta-analysis about pharmacist interventions in hypertension have revealed that interventions performed more often than once a month tended to be more effective than interventions performed less than once a month^28^. The same meta-analysis included trials outside of remote interventions, thus differing from the present study. However, our results were consistent with the previous study findings in that pharmacist interventions were useful for improving SBP among people with hypertension. Although our study did not evaluate the number of follow-ups performed, we saw that scheduling regular follow-up decreased SBP more effectively than scheduling follow-up as needed. Such a finding is novel and supports implementation of intentionally scheduling remote follow-ups by pharmacists for patients with hypertension. Regular follow-up with expected resource needs and devoted appointment time might benefit both patients and clinicians to achieve the treatment goals of chronic diseases such as hypertension.

In the present study, remote interventions by pharmacists using only telephone were not inferior to those using at least one other communication tool such as web communications or text messages. A network meta-analysis comparing the antihypertensive effects of interventions using various communication tools such as telephones, websites, Short Message Service (SMS) and smartphone- application software (apps) found that the combination of two or more tools was most effective, though the second most effective group used the telephone alone in lowering SBP^29^. The interventions in this meta-analysis were not delivered solely by pharmacists as in our study. In the present study, it was not possible to compare different types of communication tools because only two cohorts were formed due to small sample size, and one of the groups contained various types of communication tools used by pharmacists. But the result that remote interventions using telephone only were not inferior to those using other communication tools in lowering SBP was consistent with past study findings. In other words, pharmacists who do not have communication tools other than the telephone can provide effective interventions for hypertension patients if they follow-up regularly according to the planned schedule. At the same time, telephone calls can be inconvenient for some patients because they must be available at the time of the call. Although there might be a time lag from sending a message to checking and responding to the message, smartphone apps, emails, or text messages may be more convenient ways to communicate. Non-telephone tools were used for remote follow-up in three studies, but none of these examined age-related differences on intervention effectiveness. Therefore, it was not possible to assess statistically the impact of age-related differences in ability to use communication tools on intervention effectiveness except for telephone. Further studies are needed to evaluate the ability to use various communication tools by older patients, and when pharmacists follow up remotely with patients having hypertension, it would be best to use communication tools that fit the individual patient’s lifestyle and ability best.

Previous meta-analyses comparing pharmacist-led interventions and collaborative interventions by pharmacists and other healthcare professionals for hypertension patients showed that pharmacist-led interventions tended to be more effective. However, these intervention methods were not limited to remote follow-ups, as in the present study^28^. Thus, our study findings add to the existing evidence on pharmacist interventions and are consistent with previous study findings in which pharmacist-led interventions were effective regardless of collaboration with other professionals. However, the quality and frequency of pharmacists’ reports to physicians in PCC were not evaluated in our study. To enhance the effects of remote follow-up, the quality and timing of communication with physicians may be evaluated in future studies.

One of the limitations of the study is that the research outcome was limited to SBP as diastolic BP (DBP) values were not available in all included studies. The timing of SBP measurements differed among the included studies. The DBP outcome and timing of BP measures should be evaluated in future studies. We could not evaluate the impact of comorbidities and age on the intervention because the SBP of patients with each comorbidity was not available from the included RCTs. In AFC, follow-up was carried out as needed, so it is possible that not all subjects received remote follow-up by pharmacists during the study period. The impact of COVID-19 could not be assessed in the current study because study periods indicated by the included articles were prior to the beginning of the COVID-19 pandemic.

## Conclusion

The present meta-analysis revealed that remote follow-up by pharmacists reduced SBP in patients with hypertension. In addition, regularly scheduled follow-up contributed to the success of remote follow-up compared with as needed follow-up. Higher quality studies are needed to identify the ideal combination of remote follow-up communication tools and methods that affect BP reduction.

## Materials and methods

### Data search and study selection

Based on Preferred Reporting Items for Systematic Reviews and Meta-Analyses (PRISMA) 2020 guideline^30^ (PRISAMA2020 check list: Supplementary Table 3, PRISAMA2020 abstract check list: Supplementary Table 4), the search was conducted using PubMed/Medline, Embase, and Cochrane Library from June to July 2023. We searched for available articles published between October 1982 and June 2023. Searched terms included “pharmacist,” “hypertension,” and “randomized controlled trial” (Terms used for the search and the PubMed search details are shown in Supplementary Table 5). The search strategy is summarized in Fig. 1. Duplicate records, non-English records, conference reports, reviews, meta-analyses, research protocols, and reports about non-RCT trials were excluded from resulting records. Two reviewers independently assessed the articles for eligibility and data extraction and resolved disagreements by consensus. The inclusion criteria for the meta-analysis were as follows: (a) RCTs involving hypertension patients with or without coexisting chronic conditions (e.g., diabetes, CKD, CVD, stroke, human immunodeficiency virus [HIV] infection), (b) pharmacists using remote communication tool(s) to follow up with subjects during the intervention period, (c) studies reporting SBP at baseline and during the intervention period. Extracted data included study setting, characteristics of participants, intervention periods, types of remote communication tools used (e.g., telephone, text message, web communications, mail), number of study subjects and SBP. Finally, we excluded studies with inadequate values for Standard Deviation (SD) or 95% CI.

### Outcome and data analysis

We selected SBP as the primary outcome of this meta-analysis because a reduction in SBP lowered the risk of cardiovascular events in the previous research. For instance, a 5 mmHg and 10 mmHg reduction in SBP decreased the risk of developing cardiovascular events by 10% and 20%, respectively^3,5^. The subjects receiving remote follow-up were categorized into the “IG”, while those receiving usual in-person follow-up categorized into the “CG”. We conducted the meta-analysis to elucidate the regularity of follow-up, the types of communication tools, and communication with physicians. We used the software Review Manager version 5.4 (The Cochrane Collaboration, London, UK). The mean difference, SD, and 95% CI were used to estimate effects. The calculator of Review Manager was used when it was necessary to calculate SD from the mean value of SBP and 95% CI. If the differences between two 95% CI values and the mean value differed by 0.1 from each other, the smaller value of 95% CI was adopted. The meta-analysis was undertaken using the random-effects model with the results presented in a forest plot. Statistical heterogeneity was evaluated by the I^2^ statistic. Since the present study was a literature review and meta-analysis of published data, no ethical or human subject protection evaluation was required.

### Assessment of risk of publication bias

Two reviewers independently assessed the risk of bias with any disagreement resolved by consensus. Cochrane's ROB2 tool was used to assess the risk of bias^31^. This tool contains the following six assessment domains: (a) randomization process; (b) deviation from the intended interventions; (c) missing outcome data; (d) measurement of the outcome; (e) selection of the reported results; and (f) overall risk of bias. Each domain was ranked “low risk of bias,” “some concerns,” or “high risk of bias.” In addition, a funnel plot was constructed and Egger’s test^32^ and trim-and-fill analysis^33^ were conducted to detect the presence of potential publication bias in this random-effects meta-analysis model (Restricted Maximum Likelihood method) using statistical software Stata /MP version 18.0 (Stata Corp LLC, College Station, USA).

### Sensitivity analysis

To assess data robustness, a sensitivity analyses was conducted by using Review manager. In the sensitivity analyses, we evaluated whether the results were affected by (1) excluding the study with the highest number of participants, (2) excluding the study in which the intervention reduced SBP the most, (3) excluding studies with high bias, (4) changing measurement time from the final point to the earlier time of intervention period (There were two studies in that SBP was measured at multiple times during the intervention period, and SBP was measured twice in both studies during the intervention period) and (5) changing from the random-effects model to the fixed-effect model.

### Subgroup analysis

#### Remote follow-up success factor

We conducted three subgroup analyses to identify factors contributing to success of remote follow-up by pharmacists for BP improvement.

#### Regularity of follow-up

Thirteen studies were divided into two groups according to the following criteria. The studies having specific contact time or frequency of interventions were classified as “RFC”, while the studies without specific contact time or frequency of interventions were classified as “AFC”. RFC also contained the studies in which they conducted both regular and as needed follow-up.

#### Types of communication tools

Thirteen studies were divided into two groups according to the following criteria. The studies that used telephone only were classified as “TCC”, while the studies that used various communication tools were classified as “OCC” (Supplementary Table 1). OCC also contained the studies in which both telephone and other communication tools were used.

#### Communication with physician

Thirteen studies were divided into two groups according to the following criteria. In “PCC”, there were descriptions in the articles that pharmacists reported information from the patient encounters or their recommendations to physicians, while there was no description about them in the “NPC”.

### Supplementary Information

Supplementary Information.

## Data Availability

All data generated or analyzed during this study are included in this published article.

## References

[1] Lewington S, Clarke R, Qizilbash N, Peto R, Collins R (2002). Age-specific relevance of usual blood pressure to vascular mortality: A meta-analysis of individual data for one million adults in 61 prospective studies. Lancet.

[2] Lawes CM (2003). Blood pressure and cardiovascular disease in the Asia Pacific region. J. Hypertens..

[3] Ettehad D (2016). Blood pressure lowering for prevention of cardiovascular disease and death: A systematic review and meta-analysis. Lancet.

[4] Thomopoulos C, Parati G, Zanchetti A (2018). Effects of blood pressure-lowering treatment on cardiovascular outcomes and mortality: 14-effects of different classes of antihypertensive drugs in older and younger patients: overview and meta-analysis. J. Hypertens..

[5] Canoy D (2022). How much lowering of blood pressure is required to prevent cardiovascular disease in patients with and without previous cardiovascular disease?. Curr. Cardiol. Rep..

[6] Zhou B (2021). Worldwide trends in hypertension prevalence and progress in treatment and control from 1990 to 2019: A pooled analysis of 1201 population-representative studies with 104 million participants. Lancet.

[7] Burnier M, Egan BM (2019). Adherence in hypertension. Circ. Res..

[8] Christensen A, Osterberg LG, Hansen EH (2009). Electronic monitoring of patient adherence to oral antihypertensive medical treatment: A systematic review. J. Hypertens..

[9] Wetzels G (2006). Determinants of poor adherence in hypertensive patients: Development and validation of the “Maastricht Utrecht Adherence in Hypertension (MUAH)-questionnaire”. Patient Educ. Couns..

[10] Wetzels GE (2007). Electronic monitoring of adherence as a tool to improve blood pressure control. A randomized controlled trial. Am. J. Hypertens..

[11] Gupta P (2017). Biochemical screening for nonadherence is associated with blood pressure reduction and improvement in adherence. Hypertension.

[12] Uhlig K, Patel K, Ip S, Kitsios GD, Balk EM (2013). Self-measured blood pressure monitoring in the management of hypertension: A systematic review and meta-analysis. Ann. Intern. Med..

[13] Tucker KL (2017). Self-monitoring of blood pressure in hypertension: A systematic review and individual patient data meta-analysis. PLoS Med..

[14] Green BB (2008). Effectiveness of home blood pressure monitoring, Web communication, and pharmacist care on hypertension control: A randomized controlled trial. JAMA.

[15] Jackson IL, Ukwe CV (2021). Clinical outcomes of pharmaceutical care intervention in HIV positive patients with hypertension: A randomized controlled study. J. Clin. Pharm. Ther..

[16] Nakanishi M (2021). Impact of pharmacist intervention for blood pressure control in patients with chronic kidney disease: A meta-analysis of randomized clinical trials. J. Clin. Pharm. Ther..

[17] Carter BL (2009). Physician and pharmacist collaboration to improve blood pressure control. Arch. Intern. Med..

[18] Carter BL (2015). Cluster-randomized trial of a physician/pharmacist collaborative model to improve blood pressure control. Circ. Cardiovasc. Qual. Outcomes.

[19] Magid DJ (2011). A multimodal blood pressure control intervention in 3 healthcare systems. Am. J. Manag. Care.

[20] Margolis KL (2013). Effect of home blood pressure telemonitoring and pharmacist management on blood pressure control: A cluster randomized clinical trial. JAMA.

[21] Solomon DK (1998). Clinical and economic outcomes in the hypertension and COPD arms of a multicenter outcomes study. J. Am. Pharm. Assoc. (Wash).

[22] Li Y (2021). Effects of pharmacist intervention on community control of hypertension: A randomized controlled trial in Zunyi, China. Glob. Health Sci. Pract..

[23] Li N (2023). Impact of medication therapy management (MTM) service model on multi-morbidity (MMD) patients with hypertension: A pilot RCT. BMC Geriatr..

[24] Mathews AS, Kumari S (2022). Impact of pharmacist LED hypertension management. Asian J. Pharm. Clin. Res..

[25] Scala D (2018). Are you more concerned about or relieved by medicines? An explorative randomized study of the impact of telephone counseling by pharmacists on patients' beliefs regarding medicines and blood pressure control. Patient Educ. Couns..

[26] Stewart K (2014). A multifaceted pharmacist intervention to improve antihypertensive adherence: A cluster-randomized, controlled trial (HAPPy trial). J. Clin. Pharm. Ther..

[27] Zaragoza-Fernandez MP, Gastelurrutia MA, Cardero M, Martinez-Martinez F (2012). Intensive two-month intervention on diet and lifestyle in uncontrolled hypertensive patients in a community pharmacy. Latin Am. J. Pharm..

[28] Santschi V (2014). Improving blood pressure control through pharmacist interventions: A meta-analysis of randomized controlled trials. J. Am. Heart Assoc..

[29] Cavero-Redondo I (2021). Comparative effect of eHealth interventions on hypertension management-related outcomes: A network meta-analysis. Int. J. Nurs. Stud..

[30] Page MJ (2021). The PRISMA 2020 statement: An updated guideline for reporting systematic reviews. BMJ.

[31] Sterne JAC (2019). RoB 2: A revised tool for assessing risk of bias in randomised trials. BMJ..

[32] Egger M, Smith GD, Schneider M, Minder C (1997). Bias in meta-analysis detected by a simple, graphical test. BMJ.

[33] Duval S, Tweedie R (2000). Trim and fill: A simple funnel-plot-based method of testing and adjusting for publication bias in meta-analysis. Biometrics.

